# Unusual Foreign Body in the Middle Ear: Surgical Removal of a Live Ant Entering the Tympanic Cavity Through an Ear Drum Perforation

**DOI:** 10.3390/diagnostics14222530

**Published:** 2024-11-12

**Authors:** Peter Kiss, Jakob Pock, Michael Habenbacher, Emanuel Maitz, Angelika Lang, Katharina Walla, Alexandros Andrianakis

**Affiliations:** Department of Otorhinolaryngology, Medical University of Graz, Auenbruggerplatz 26, 8010 Graz, Austria

**Keywords:** tympanon, tympanic membrane perforation, ear surgery, endoscopic tympanotomy, ear disease

## Abstract

This case report details the unusual occurrence of a live ant invading the middle ear cavity through a pre-existing perforation in the tympanic membrane of a 42-year-old female patient. She presented to an outpatient clinic with symptoms of sudden-onset tinnitus (“ringing”) and a foreign body sensation in her left ear. Otomicroscopy revealed an oval-shaped perforation in the posterior part of the left tympanic membrane, through which a dark, moving foreign object was observed in the middle ear. The object was identified as a live ant. Initial attempts to remove the insect under local anesthesia were unsuccessful, necessitating the patient’s admission for surgery. Under general anesthesia, an endoscopic tympanotomy was performed, and the ant was successfully removed without complications. The patient recovered and was discharged the following day. At her follow-up appointment, she remained symptom-free. This case highlights the rare yet possible occurrence of live foreign bodies entering the middle ear through tympanic perforations and the need for timely surgical intervention to prevent complications.

Figure 1 shows the initial otomicroscopic view of the left ear. The pre-known oval-shaped ear drum perforation can be seen in the posterior part of the tympanic membrane. Behind the anterior part of the eardrum, a small, dark foreign body was detected, identified as an insect, specifically a live ant, which had entered the middle ear cavity through the perforation. The presence of the live insect was confirmed as it exhibited movement upon examination.

Figure 2 shows the intraoperative view during an endoscopic tympanotomy performed under general anesthesia. A typical tympanomeatal flap was raised to provide better access to the middle ear cavity. The foreign body, a live ant, was located and carefully extracted. The procedure was completed without any intraoperative complications. The patient experienced no adverse effects and was discharged the day after surgery. During the follow-up examination, no residual symptoms were reported, and the ear healed appropriately, without signs of infection or damage from the procedure.

Foreign bodies in the external ear canal are relatively common, but their presence in the middle ear is extremely rare. Most cases of middle ear foreign bodies involve silicone material left during ear mold impressions, as documented by AL Zaabi et al. in their review of 42 published cases [1]. Other cases include metallic foreign bodies, typically resulting from welding injuries, with only four cases reported in the literature [2,3,4,5]. Cases of live insects in the middle ear have been documented, specifically otomyiasis. A recent review identified 16 cases of fly larvae affecting the middle ear. These instances are exceedingly rare, with limited case series available to guide clinical management [6]. On the other hand, live insects entering the external auditory canal are much more common. According to large-cohort studies, insects account for 10–50% of foreign bodies in the external ear canal [7,8,9,10,11,12,13]. Supiyaphun et al. reported a rare case of a mature termite who entered the auditory canal and perforated the eardrum to enter the tympanic cavity [14]. Our particular case of a live ant entering the middle ear through a pre-existing tympanic membrane perforation represents a unique clinical scenario. It underscores the rare yet possible occurrence of live insects entering the middle ear through pre-existing tympanic membrane perforations, emphasizing the importance of timely surgical intervention to prevent complications. In cases of sudden tinnitus and ear fullness, a thorough ear examination is recommended, especially in patients with eardrum perforations, to avoid missing rare conditions like this.

## Figures and Tables

**Figure 1:**
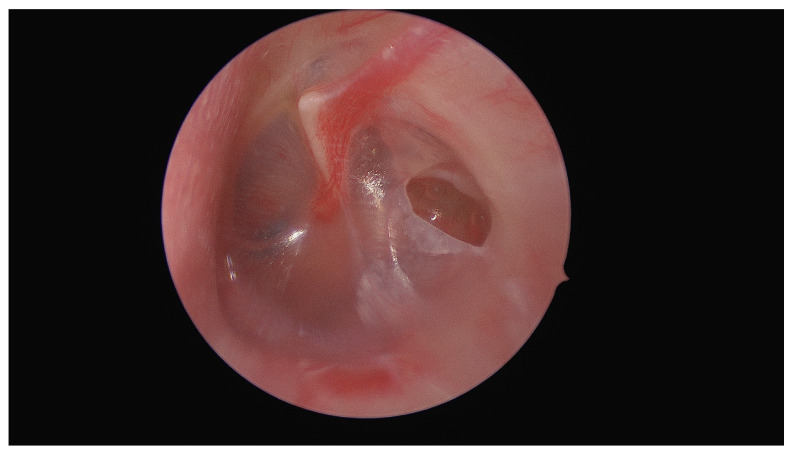
A 42-year-old female patient with a known pre-existing perforation of the left tympanic membrane presented to the outpatient clinic of the Department of Otorhinolaryngology, Medical University of Graz, with symptoms of sudden-onset, intermittent tinnitus (like a “scratchy ringing”) and a sensation of fullness in the left ear. She reported no hearing loss or vertigo.

**Figure 2:**
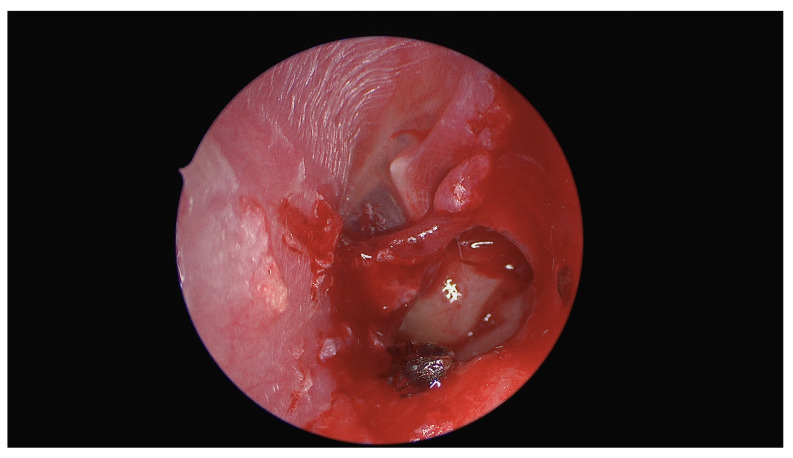
Initial attempts to remove the insect under local anesthesia in the outpatient setting were unsuccessful. For local anesthesia, the whole external ear canal was filled with a ribbon gauze soaked in lidocaine to prevent the anesthetic from entering the middle ear cavity, as lidocaine is ototoxic. This approach did not have any effect, even transient, on the facial nerve or vestibulocochlear function. The patient was subsequently directly hospitalized to undergo an operation under general anesthesia, without any topical treatment attempts prior surgery.
